# Research priority setting in UK podiatric surgery

**DOI:** 10.1186/s13047-023-00629-9

**Published:** 2023-06-02

**Authors:** Lesley Posmyk, Robyn L. Carter-Wale, Kerry Clark, Lorna Donson, Jill Halstead, Natalie Lennox, Helen Milnes

**Affiliations:** 1 Department of Podiatric Surgery, North Tees & Hartlepool NHS Foundation Trust, Hartlepool, UK; 2 Department of Podiatric Surgery, Central London Community Healthcare NHS Trust, London, UK; 3 Department of Podiatric Surgery, University Hospitals Birmingham NHS Foundation Trust, Birmingham, UK; 4 Department of Podiatric Surgery, Wye Valley NHS Trust, Hereford, UK; 5 Department of Podiatry, Leeds Community Healthcare NHS Trust, Leeds, UK

**Keywords:** Research strategy, Research plan, Podiatric surgery, Foot surgery, Research priorities

## Abstract

Evidence-based practice provides the foundation for high quality patient care, and in the NHS, research is seen as vital to enable service transformation and improve outcomes. Research is one of the four pillars of enhanced and advanced clinical practice and is therefore a fundamental part of podiatric surgery services. In order to meet the UK health research strategies, the most recent being ‘Saving and Improving Lives: The Future of UK Clinical Research Delivery’ (2021), the Faculty of Podiatric Surgery in the UK agreed to support the development of research priorities in order to inform a future research strategy.

The Podiatric Surgery Research Strategy Group was set up and embarked on a project with the aim of engaging its members in formulating and agreeing national research priorities. The initial stage included a national research scoping survey to identify key themes, topic, and research questions. The final stage consisted of developing and enabling a live consensus vote conducted at the 2022 national Faculty of Podiatric Surgery Conference. At the end of the vote, the top five research topics that met the agreement criteria were: 1. Surgical treatment – forefoot, 2. Patient reported outcome measures, 3. Post-operative management, 4. Surgical treatment – midfoot and 5. Service delivery. The top five research questions that met the criteria were1. How does quality of life improve following elective foot surgery? 2. How does podiatric surgery benefit the health of the population? 3. How does podiatric surgery benefit the health of the population in the at-risk foot? 4. What is the most effective Lapidus fixation option? and 5. What is the benefit of utilising PASCOM-10 to improve large scale outcome data? These will inform the initial UK podiatric surgery research priorities in the next three to five years.

**Supplementary Information:**

The online version contains supplementary material available at 10.1186/s13047-023-00629-9.

## Background

Podiatric surgery involves the assessment, diagnosis, and surgical management of foot and ankle pathologies [1]. There is a paucity of robust long-term research using validated outcome measures within the field of podiatric surgery in the UK. As a sub-specialism within podiatry, it was noted that there was no cohesive research strategy for podiatric surgery in line with national research policies [2–4] and agendas [5]. In addition, research, leadership, and education are all pillars of advanced practice, and are paramount to the development of podiatric surgery specialisms within the wider Podiatry profession [6]. To address this gap, a Podiatric Surgery Research Strategy Group (PSRSG) was formed in 2020. This group, consisting of podiatric surgeons and researchers, aimed to determine a set of foot and ankle surgery research priorities for Podiatric Surgery in the UK in order to inform a long-term research strategy. A detailed research priority setting process has recently been undertaken for foot and ankle surgery including patients and carers, physiotherapists, podiatrists and members of the British Orthopaedic Foot and Ankle Society (BOFAS) in association with the James Lind Alliance [7]. The BOFAS priority setting focused on surgical treatments and injections, outcomes, and post treatment aftercare but excluded multiple categories including: limb salvage, diabetes related foot pathology, pre-operative considerations, conservative management, and diagnostics. To represent the professional group, this priority setting project did not exclude any categories and aimed to cover the full scope of Podiatric Surgery practice.

## Methods

A modified Delphi approach was used to identify research questions and provide consensus on what the top research priorities should be. This approach was developed in two stages and a total of five rounds. The initial stage included two rounds to synthesise data for consensus, using a scoping survey and assessment of research results to identify and group research themes, topics, and individual questions. Stage two included three rounds of live consensus voting at a national professional conference.

## Stage 1

### Round 1: scoping survey

The PSRSG devised a survey using Microsoft Forms (Additional file 1 shows the scoping survey questions), which fellows of the Faculty of Podiatric Surgery of the Royal College of Podiatry (RCPod) were invited to complete online in October 2021. The survey was sent centrally by the RCPod to the entire professional membership group, which included 88 registered surgeons working in centres across England, Wales, and Scotland. The purpose of the scoping exercise was to understand the current research being undertaken nationally within podiatric surgery, identify research themes across multiple departments, highlight gaps in research, and identify any barriers to research. The survey was completed voluntarily and anonymously. Prospective survey participants were advised that the information collected would be used to further develop and support research, and were given two weeks to complete the survey. To ensure answers were not duplicated, fellows were asked to nominate one person to complete the survey on behalf of the whole department if they were part of a larger team. All fellows were invited to consider being part of the PSRSG.

#### Data collection

There was a 45% response rate with results received by a RCPod representative who collated the data and provided the information to the PSRSG.

### Round 2: formulation of themes, topics, and questions

Over the following 4 months the PSRSG met. The information obtained from the survey was reviewed and any areas of duplication were amalgamated to avoid repetition. Where proposed research priorities were broad or vague, these were reframed into research questions. Due to the high number of research questions (*n* = 65), the PSRSG grouped these according to naturally aligned topics and an overarching theme was identified. This structured the data into four main themes, with multiple topics and questions, outlined below (see Table 1 for the full details):

1. Health economics and service delivery: 3 topics and 11 questions
2. Patient satisfaction and patient reported outcome measures: 2 topics and 8 questions
3. Treatment: 8 topics and 33 questions
4. Limb salvage surgery: 4 topics and 13 questions

**Table 1:** Themes, topics and research questions identified from scoping survey

Topic	Question
**Theme: Health economics and service delivery**	
Service Delivery	Is Podiatric Surgery efficient and cost effective compared to other providers of foot surgery?
	What methods of service promotion are most effective in Podiatric Surgery?
	Promoting our discipline: what is the understanding of Podiatric Surgery by other healthcare professionals? How do we raise awareness?
	What is the place of Podiatric Surgery in mainstream medical care?
	What has been the impact of independent prescribing on Podiatric Surgery?
Population Health	What is the recurrence rate of hallux valgus following correction via osteotomy?
	How does Podiatric Surgery benefit the health of the population?
	What are the demographics of patients accessing Podiatric Surgery services and do they represent all groups in the local community?
Covid-19	What is the incidence of post-surgery venous thromboembolism in relation to Covid-19 vaccine in Podiatric Surgery?
	What role has Podiatric Surgery played during the pandemic?
	What operational changes to Podiatric Surgery have occurred during the pandemic?
**Theme: Patient satisfaction and patient reported outcome measures**	
Patient satisfaction	What is the effect of hallux valgus deformity on quality of life?
	Does hallux valgus surgery reduce falls in elderly patients?
	How does quality of life improve following elective foot surgery?
	What are the patient experiences of day case foot surgery?
Patient reported outcome measures	Is the Manchester Oxford Foot Questionnaire valid in elective foot surgery other than hallux valgus correction?
	Are the Short-form -36 and EuroQol-5D valid in elective foot surgery?
	What is the benefit of utilising PASCOM-10 (National audit database) to improve large scale outcome data?
	How does the patient’s verbal report of surgical success relate to quantitative patient reported outcomes measures?
**Theme: Treatment**	
Conservative treatment	Steroid injection therapy – short-, medium-, and long- term outcomes
	Is there a place for platelet-rich plasma in end-stage osteoarthritis management?
	Is hyaluronic acid, platelet-rich plasma, or steroid injection therapy most effective in 1^st^metatarsophlanageal joint osteoarthritis?
Pre-operative assessment	Is there a place for prehabilitation clinics prior to hallux valgus correction?
	Should we routinely be completing toe pressures on all elective surgery patients? Do toe pressures in elective cases accurately indicate wound healing potential?
	Does routine toe pressure assessment improve early diagnosis of vascular issues prior to elective foot surgery?
	Antibiotic prophylaxis in elective foot surgery: is it required? What modality is best? Risk stratification tool?
	Venous thromboembolism in elective foot surgery
	What is the most appropriate peri-operative management of immunosuppressive medications in elective foot surgery?
Surgical treatment-forefoot	Deep transverse ligament release vs neurectomy: A comparative study
	What is the consensus on neuroma surgery: plantar vs dorsal incision, single vs double?
	What are the latest techniques in forefoot surgery?
	Randomised study of hammer toe fusion versus arthroplasty
	Review of long-term outcomes of implants (E.g., 1^st^metatarsophalangeal joint, interphlex, proximal interphalangeal joint)
	Minimally invasive surgery versus open lesser metatarsal surgery-what are the outcomes?
Surgical treatment-midfoot	What is the most effective Lapidus fixation option?
	What are the financial implications of different fixation techniques for Lapidus?
	Is the trephine grafting technique superior to traditional joint preparation in midfoot fusion?
Surgical treatment-rearfoot	What are the advancements in rearfoot surgery?
	What is the success of Achilles Tendon lengthening in improving forefoot pain?
	What is the most effective fixation method for Talonavicular joint fusion?
Post-operative management	Review of post-operative regimen for procedures-what’s the consensus throughout the profession?
	What post-operative protocols are used following surgery (procedure specific), and do they affect patient reported outcome measures and time to recovery?
	Do post-operative range of motion exercises following hallux valgus correction improve patient outcomes?
	What is the most effective strategy for reducing digital swelling post-operatively?
	What are the best modalities for reducing post-operative scarring?
Impact factors	Does Vitamin D affect union rates post-operatively: what are the thresholds?
	Does smoking directly impact surgical outcomes in foot surgery?
	Does the use of a bone stimulator improve the speed of bony union following foot and ankle fusions?
Getting it right first time (GIRFT)	Is the World Health Organisation checklist fit for purpose?
	What patient safety tools are in use in Podiatric Surgery, and have they reduced errors?
	Minimising risk: does the imaging report match the request? Is it reducing costs? Are imaging modalities being used appropriately?
	Are current thoughts regarding common risks of surgery true, or are they outdated or incorrect?
**Theme: limb salvage surgery**	
Cost-efficiency	What are the short-, medium-, and long- term benefits of our involvement in limb salvage surgery- does it help? Does it save money? What quantifiable data can we publish to develop this arm of the profession?
	Is our involvement in limb salvage surgery beneficial to patients and financially favourable to trusts: a long-term study of outcomes and finances?
	What models are currently being used in the management of the diabetic foot? Is one more cost effective than the other and do they affect the patient pathway and outcomes?
Multi-disciplinary Team	Advancements in the diabetic foot-working with vascular in an acute setting?
	What is the understanding of Podiatric Surgery in the wider multidisciplinary team?
	Do we meet the national criteria?
Surgery / Treatment	Do variations in peri-operative management affect outcomes in the diabetic foot?
	What are the multi-centre outcomes of Podiatric Surgery in limb salvage of the diabetic foot?
	Elective prophylactic and curative approaches in limb salvage surgery, how do we compare our data using PASCOM-10 as a tool?
Patient experience	What is the psychological impact of diabetic foot ulceration and lower limb amputation?
	How does Podiatric Surgery benefit the health of the population in the at-risk foot?
	What is the patient experience of Podiatric Surgery in the management of their diabetic foot ulcer?
	Did Covid-19 delay patients seeking treatment for diabetic foot ulceration and how has this affected prognosis and outcomes?

## Stage 2: consensus voting

### Study design

The themes, topics, and research questions which were developed following the initial survey were presented at the conference. There were three rounds of voting in total to decide the highest priority theme, topic, and question. The software utilised for the voting was Slido, a smartphone App (Cisco Systems 2022 [Webex], Slovakia), which is interactive and allows anonymous participation. The PSRSG pre-determined that for strong consensus to be obtained a high level of agreement at 75% must be achieved, with a moderate agreement set from 74 to 65% and low agreement to between 64 to 55%, with a minimum threshold of 55% to be attained. 75% is the most cited threshold for agreement within Delphi consensus studies [8], therefore was deemed appropriate for high agreement within this study.

### Participants

To ensure wide-ranging representation, the annual Faculty of Podiatric Surgery conference in March 2022 was selected to allow purposive sampling. This was attended by podiatric surgeons, podiatrists, podiatry students, academics, researchers, and other healthcare professionals. Formal written consent was not deemed necessary as all voting participation was voluntary and anonymity was maintained. The delegates were advised beforehand that by engaging in the live vote they were providing their consent to participate in the consensus process and publication of the results.

### Data collection

#### Round 3: research themes

The third round ranked the research themes (Table 2) in order of preference with one being the most important and four being the least important. The voting was conducted in real time and the results determined the subsequent topics presented in round four. The results were received via the Slido App, which was monitored by two members of the PSRSG, who modified the topics presented in round four according to the live voting scores.

**Table 2:** Research themes for ranking

**Theme**	**Examples**
Health economics and service delivery	Demonstrating the value of podiatric surgery, impact on population health, efficiency
Limb salvage surgery	Treatment, multi-disciplinary team working, patient experiences, outcomes
Treatment	Surgical outcomes (inc. long-term outcomes), procedure specific projects, peri-operative management, getting it right first time
Patient satisfaction/ Patient reported outcome measures	Patient reported outcome measures, validity of outcome measures in podiatric surgery, patient experience, quality of life impact

#### Round 4: research topics

In the fourth round each participant was asked to vote on the topic they considered to be of the highest priority within the overarching research theme (Table 1). This was scored as a percentage vote of the total participants who participated in this round.

#### Round 5: research questions

The fifth round presented research questions for the topics that had reached the minimum threshold from the voting in round four. Failure of the topic to reach the pre-determined minimum threshold resulted in exclusion of the research questions in this round. Participants were again asked to vote for their highest priority question, and was scored as a percentage vote of the total participants who participated in this round.

### Descriptive analysis

For Delphi stage two, round three, theme ranking was calculated by assigning a points value for each rank position (1^st^ place = 40 points, 2^nd^ place = 20 points, 3^rd^ place = 10 points, 4^th^ place = 5 points), then multiplying this by the number of times it was placed in a particular rank. These scores were totalled for each theme to provide a score for proportional ranking.

For rounds four and five, agreement rates were expressed as a percentage of participants who voted in each round. Agreed thresholds of consensus were pre-set by the PSRSG as: High agreement =  ≥ 75%; Moderate agreement = 74% to 65%- and a Low agreement = 64% to 55%. Any values < 55% did not meet the agreement criteria.

## Results

One hundred one delegates participated in the research priorities live consensus voting, with the same 101 delegates consistently voting anonymously within each round of ranking. This comprised of accredited podiatric surgeons (57%), other healthcare professionals (16%), podiatric surgery trainees (13%), student podiatrists (undergraduate and postgraduate, 9%), and observers (commercial and sponsors, 5%). Whilst commercial observers / sponsors had the opportunity to vote, the proportion of voters within this category of non-healthcare professionals is comparable to that reported by the BOFAS James Lind research priorities project. The authors recognise the potential portal for commercial bias; however, this group accounted for a small percentage of the participating group and none of the questions were directly related to a specific product or company.

Round three voting ranked the themes from highest to lowest priority as: Health economics and service delivery; Treatment; Limb salvage surgery; and Patient satisfaction/Patient reported outcome measures.

Subsequent voting in rounds four and five with the themes, topics and research questions are shown in Table 3 along with the associated consensus agreement percentages.

**Table 3:** Themes, topics, and research questions with agreement percentages

**Topic**	**Rank**	**Question**	**Agreement reached (%)**
**1**^**st**^**: Health economics and service delivery**			
Service delivery	1^st^ 54%	Is podiatric surgery efficient and cost effective compared to other providers of foot surgery?	41
		Promoting our discipline: what is the understanding of podiatric surgery by other healthcare professionals? How do we raise awareness?	27
		What is the place of podiatric surgery in mainstream medical care?	23
		What has been the impact of independent prescribing on podiatric surgery?	6
		What methods of service promotion are most effective in podiatric surgery?	3
Population health	2^nd^ 41%	How does podiatric surgery benefit the health of the population?	73^**^
		What are the demographics of patients accessing podiatric surgery services and do they represent all groups in the local community?	14
		What is the recurrence rate of hallux valgus following correction via osteotomy?	13
Covid-19	3^rd^ 5%	What operational changes to podiatric surgery have occurred during the pandemic?	42
		What role has podiatric surgery played during the pandemic?	31
		What is the incidence of post-surgery venous thromboembolism in relation to Covid-19 vaccination in podiatric surgery?	28
**2**^**nd**^**: Treatment**			
Surgical treatment: forefoot	1^st^ 78%	Review of long-term outcomes of implants (e.g., 1^st^ metatarsophalangeal joint, interphlex, proximal interphalangeal joint)	27
		Minimally invasive surgery versus open lesser metatarsal surgery – what are the outcomes?	26
		What are the latest techniques in forefoot surgery?	20
		Randomised study of hammer toe fusion version arthroplasty	17
		Deep transverse ligament release vs. neurectomy: A comparative study	6
		What is the consensus on neuroma surgery: plantar versus dorsal incision, single versus double?	4
Post-operative management	2^nd^ 64%	What post-operative protocols are used following surgery (procedure specific), and do they affect patient reported outcome measures and time to recovery?	46
		Review of post-op regimen for procedures – what is the consensus throughout the profession?	23
		Do post-operative range of motion exercises following hallux valgus correction improve patient outcomes?	16
		What is the most effective strategy for reducing digital swelling post-operatively?	8
		What are the best modalities for reducing post-operative scarring?	6
Surgical treatment: midfoot	3^rd^ 62%	What is the most effective Lapidus fixation option?	62^*^
		Is the trephine grafting technique superior to traditional joint preparation in midfoot fusion?	31
		What are the financial implications of different fixation techniques for Lapidus?	7
GIRFT	4^th^ 54%	Are current thoughts regarding common risks of surgery true, or are they outdated and incorrect?	51
		What patient safety tools are in use in podiatric surgery, and have they reduced errors?	29
		Minimising risk: does the imaging report match the request? Is it reducing costs? Are imaging modalities being used appropriately?	18
		Is the World Health Organisation checklist fit for purpose?	3
**3**^**rd**^**: Limb Salvage**			
Surgery / Treatment	1^st^ 41%	What are the multi-centre outcomes of podiatric surgery in limb salvage of the diabetic foot?	45
		Elective prophylactic and curative approaches in limb salvage surgery, how do we compare our data using PASCOM-10 (National database) as a tool?	29
		Do variations in peri-operative management affect outcomes in the diabetic foot?	26
Cost-efficiency	2^nd^ 26%	What are the short-, medium-, and long-term benefits of our involvement in limb salvage surgery-does it help? Does it save money? What quantifiable data can we publish to develop this arm of the profession?	50
		Is our involvement in limb salvage surgery beneficial to patients and financially favourable to Trusts: a long-term study of outcomes and finances	36
		What models are currently being used in the management of the diabetic foot? Is one more cost-effective than the other and do they affect the patient pathway and outcomes?	15
Multi-disciplinary team	3^rd^ 25%	What is the understanding of podiatric surgery in the wider multi-disciplinary team?	52
		Advancements in the diabetic foot – working with vascular in the acute setting	44
		Do we meet the national criteria?	4
Patient experience	4^th^ 8%	How does podiatric surgery benefit the health of the population in the at-risk foot?	63^*^
		What is the psychological impact of diabetic foot ulceration and lower limb amputation	20
		What is the patient experience of podiatric surgery in the management of their diabetic foot ulcer?	14
		Did Covid-19 delay patients seeking treatment for diabetic foot ulceration and how has this affected prognosis and outcomes?	3
**4**^**th**^**: Patient satisfaction and patient-reported outcomes**			
Patient reported outcome measures	1^st^ 66%	What is the benefit of utilising PASCOM-10 to improve large scale outcomes data?	53
		Is the Manchester Oxford Foot Questionnaire valid in elective foot surgery other than hallux valgus correction?	23
		How does the patient’s verbal report of surgical success relate to quantitative PROMS?	14
		Are the Short form-36 and EuroQol-5D valid in elective foot surgery?	11
Patient satisfaction	2^nd^ 34%	How does quality of life improve following elective foot surgery?	77^***^
		What are the patient experiences of day case foot surgery?	10
		What is the effect of hallux valgus deformity on quality of life?	8
		Does hallux valgus surgery reduce falls in elderly patients?	4

^***^high agreement (≥ 75%)

^**^moderate agreement (74% to 65%)

^*^low agreement (64% to 55%)

### Highest priority theme: health economics and service delivery

There were three topics included in this theme: service delivery, population health, and Covid-19. The pre-agreed threshold level (minimum of 55%) to determine which was the highest priority topic was not reached: service delivery was the highest-ranking topic with 54%. Population health was second with 41%. The highest scoring research question within this theme was ‘how does podiatric surgery benefit the health of the population?’, which achieved high consensus score of 73%. All other questions within the theme did not reach the minimum consensus threshold.

### Second place theme: treatment

**Fig. 1:**
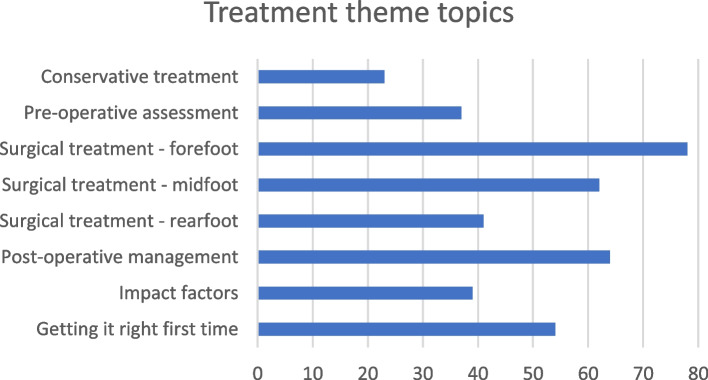
Treatment theme topics

Treatment was ranked second. There were eight topics within this theme (Fig. 1), three of which reached the minimum consensus threshold, with only one achieving high agreement: surgical management of the forefoot (78%), post-operative management (64%), and surgical management of the midfoot (62%). Of the 33 questions within this theme, only one question met the minimum threshold: ‘what is the most effective Lapidus fixation option?’ (62%). Despite the high agreement threshold being reached for the topic of forefoot surgery, the highest scoring question within this topic, demonstrated very low consensus (27%): ‘review of long-term outcomes of forefoot implants’.

### Third place theme: limb salvage surgery

Limb salvage surgery ranked third. There were four topics in this theme, none of which reached the minimum threshold. One question reached the moderate consensus threshold with 63% of the votes: ‘how does podiatric surgery benefit the health of the population in the at-risk foot?’. 41% of the delegates considered research into surgery and treatment outcomes, multicentre trials, and the use of the PASCOM-10 database in this patient cohort to be of a higher priority than cost efficiency (26%), multi-disciplinary team-working (25%), and patient experience (8%). However, none of these scored highly enough to make the final priorities list. The highest scoring questions included: ‘exploring the understanding of podiatric surgery in the wider multidisciplinary team’ (52%), ‘establishing short/long-term benefits of the involvement of podiatric surgery in limb salvage: does it save money?’ (50%), and ‘reviewing multi-centre outcomes of podiatric surgery in limb salvage of the diabetic foot’ (45%).

### Fourth place theme: patient satisfaction / patient reported outcome measures

This theme consisted of two topics, and was ranked the lowest priority theme. Despite this, it provided one of the highest individual question consensus scores: ‘how does quality of life improve following elective foot surgery?’ (77%). Of the two topics, patient reported outcome measures was considered a higher priority compared with patient satisfaction: scoring 66% and 34% respectively. However, the top-scoring question within patient reported outcome measures only achieved low consensus: ‘What is the benefit of utilising PASCOM-10 to improve large scale outcome data?’ (53%).

### Summary of highest scoring research topics and research questions

Tables 4 and Table 5 show the top five research topics and top five research questions, ranked from highest to lowest. There was one question and one theme that reached the high consensus threshold, one question which reached moderate threshold, and the remaining seven met the minimum threshold.

**Table 4:** Top five research topics based on threshold scores

**Topics**	**Summary of research identified within the topic**	**Agreement reached (%)**
1. Surgical treatment-forefoot	Surgical outcomes, fixation, minimally invasive surgery, procedure specific projects	78%^***^
2. Patient reported outcome measures	Validity of Patient reported outcome measures in elective foot surgery, PASCOM-10 (National audit system)	66%^**^
3. Post-operative management	Post-operative protocols, complications management, impact on outcomes	64%^*^
4. Surgical treatment – midfoot	Surgical outcomes, fixation, Lapidus, trephine technique	62%^*^
5. Service delivery	Efficiency of podiatric surgery, promoting podiatric surgery, factors affecting service delivery	55%^*^

^***^high agreement (≥ 75%)

^**^moderate agreement (74% to 65%)

^*^low agreement (64% to 55%)

**Table 5:** Top five research questions based on threshold scores

**Questions**	**Agreement reached (%)**
1. How does quality of life improve following elective foot surgery?	77%^***^
2. How does podiatric surgery benefit the health of the population?	73%^**^
3. How does podiatric surgery benefit the health of the population in the at-risk foot?	63%^*^
4. What is the most effective Lapidus fixation option?	62%^*^
5. What is the benefit of utilising PASCOM-10 (National audit system) to improve large scale outcome data?	53%

^***^high agreement (≥ 75%)

^**^moderate agreement (74% to 65%)

^*^low agreement (64% to 55%)

## Discussion

The aim of this study was to provide, for the first time, an agreed set of research priorities for the UK podiatric surgery profession to facilitate a long-term research strategy. This work was undertaken to meet the NHS research agenda and there was an identified gap in the four recently published foot-related research priorities from 2019 to 2022 [7, 9–11]. The initial scoping exercise identified key research themes, topics, and questions to form the consensus voting completed at the annual podiatric surgery conference. The strength of this study was the participation of 101 delegates at the national conference who have determined the future research priority questions and topics: shaping the future research strategy for the Podiatric Surgery profession.

The highest priority theme identified in the consensus voting was ‘health economics and service delivery’. Within this theme, service delivery was ranked the highest priority topic, despite this none of the questions within this topic reached the minimum agreement threshold. Interestingly, the highest scoring question came from the second-ranked topic within the theme of ‘health economics and service delivery’ and achieved high consensus: ‘How does podiatric surgery benefit the health of the population?’. Participants appeared to feel strongly that the benefits of podiatric surgery services on public health were important, especially considering this was ranked among the most highly-scoring questions overall. It is noted this theme is not included in the other foot related research prioritisation strategies [7, 9–12].

The second highest theme was ‘treatment’. Within this theme, surgical management of the forefoot was the highest priority topic and was one of only two areas within the whole priority voting to achieve high agreement levels with a score of 78%. This is unsurprising considering the prevalence of forefoot pathology in the foot and the frequency of hospital referrals [13, 14]. Although forefoot surgery was ranked the highest priority topic, none of the questions reached the pre-agreed consensus level, potentially demonstrating a broader scope of interest amongst the delegates across the individual questions included. However, another explanation could be that there were more questions to select from in the forefoot topic (six versus three to four in the other topics), therefore reducing the likelihood of agreement. Interestingly, the third-placed, midfoot topic produced the highest scoring research question of this theme: ‘what is the most effective Lapidus fixation option?’ with a score of 62%.

Limb salvage surgery ranked third in the theme priority voting, potentially reflecting the number of emerging podiatric surgery units currently involved in management of this patient cohort. There is a growing number of podiatric surgery centres that are becoming involved in the surgical management of the diabetic foot, forging relationships with other professions, and championing multi-disciplinary team working to reduce tissue loss and amputation. Encouraging multi-disciplinary working was also noted as a priority within the vascular priorities project to improve patient outcomes [11], and since the covid-19 pandemic podiatric surgeons have been increasingly recognised as key members in the limb salvage team [15, 16]. Despite this, a possible explanation for the low ranking of this theme is that not all podiatric surgery centres in the UK are undertaking limb salvage surgery at the present time. This, however, may change in the future due to the ever-increasing population with diabetes [17] and the growing recognition for podiatric surgery specialism within limb salvage [18, 19].

Collecting patient reported outcome measures are a valuable part of podiatric practice in demonstrating both the efficacy and scope of surgical intervention. PASCOM-10 is a national database used widely within podiatric surgery since May 2010. This collates a range of pre- and post-operative information. It is used by podiatric surgeons and podiatrists for assurances and governance regarding patient outcomes and patient safety. Given the wide use of PASCOM-10 and the known importance of demonstrating patient outcomes, it was interesting that this theme was voted the lowest priority. A possible explanation of this is that it may be seen as a fundamental part of day-to-day surgical practice. The highest scoring research question: ‘how does quality of life improve after foot surgery?’ is something that PASCOM-10 data does not directly collect. Another reason for quality of life scoring particularly highly compared to other themes and topics may be due to the low number of topics and questions within the theme, therefore increasing the chance of a high consensus score. The themes of patient reported outcome measures and the impact of foot surgery on population health were reflected in 4 out of the 5 top research questions. These results are in agreement with both the published research priorities on Foot and Ankle Surgery and Foot Health by James Lind Alliance, which demonstrates the importance of further research in this topic. Interestingly, it was only the top research question that demonstrated similarity with the BOFAS priority setting project, while the remainder focused on rearfoot and ankle surgery. The podiatric surgery priorities tended to rank forefoot and midfoot surgeries as high priorities, which may be explained by the scope and focus of a single professional group.

These results have provided a consensus regarding the top ten research priorities and inform the podiatric surgery research strategy. These results must be considered in the light of the following limitations: resources and time only allowed for one stage of live voting. If there had been an opportunity for a second vote, higher consensus scores may have been achieved by removing lower ranked themes, topics, and questions. Another factor to consider is that bias may have been created by the unequal grouping of questions within each theme synthesised from the scoping survey: those with fewer questions are more likely to achieve greater consensus agreement scores. A disadvantage of targeting attendees of the podiatric surgery conference is that the opinions of other specialty groups (e.g., orthotists, physiotherapists, etc.) and patients could not be included.

This project did not receive any funding, and therefore was constrained by potential costs of organising and inviting patient groups, carers, and other specialities to the consensus study. The authors recognise the limitation that people and carers who have experienced foot surgery were not included in this study and this could have influenced the initial scoping survey, the subsequent rankings and ultimately the final priorities list. As this is the first research priority setting project within Podiatric Surgery, it was felt that completing the consensus voting live during the national conference would allow the greatest opportunity to discuss the importance of the project, in addition to engaging the greatest number of professionals. This approach limited patient involvement; however, the final agreement was not reliant on the completion and return of questionnaires which typically have a lower response rate [20].

This initial consensus will provide a foundation for research within podiatric surgery, on which the priorities and strategy can be refined at future events. Working groups to address each of the top five topics and questions are in progress. Collaboration with partners at higher education institutions who provide postgraduate podiatric surgery training has begun, with the aim of embedding a strong research culture within the training programme. Involvement with the Royal College of Podiatry Research Development and Innovation Committee has been integral to progressing this project, with a view to integrating into the overall research strategy of the Royal College of Podiatry. While the initial focus of research within podiatric surgery will aim to explore the top research questions and topics identified, questions which ranked highly but that did not place in the top 10 will be made available to undergraduate podiatry students and post graduate university students studying the Masters of Science in Podiatric Surgery. This will allow the research themes to be utilised with commencement of foundation studies and post graduate studies.

## Conclusion

This consensus study has identified the key research topics and questions within the profession. It demonstrates an interest of the profession in supporting and conducting research, particularly in relation to how it benefits population health. The main areas highlighted by the highest-ranking themes, topics, and questions appear to be in understanding specific outcomes of surgical procedures, and the impact on patient quality of life. The creation and focus of these research priorities will provide a strategy that can be embedded from trainee through to consultant podiatric surgeon, appropriately channel resources, and create a positive research culture for both patient and professional benefit.

## Supplementary Information

**Additional file 1.** Faculty of Podiatric Surgery Research Survey.

## Data Availability

Data analysed during this study are available from the corresponding author on reasonable request.

## Abbreviations

PSRSG: Podiatric Surgery Research Strategy Group
RCPod: Royal College of Podiatry
BOFAS: British Orthopaedic Foot & Ankle Society
