# Supplementary material for: Research priority setting in UK podiatric surgery
Source: J Foot Ankle Res. 2023 Jun 2;16:32. doi: 10.1186/s13047-023-00629-9 (PMC10235831; doi:10.1186/s13047-023-00629-9)
Supplement: Supplementary file 1 — Additional file 1. Faculty of Podiatric Surgery Research Survey. [file 13047_2023_629_MOESM1_ESM.pdf]

Additional file 1

Faculty of Podiatric Surgery Research Survey

Qu 1. Which deanery are you part of?	
- North<br>- Central<br>- South	
**Qu 2. What is your area of practice?**	
- NHS only	Go to Qu 3
- Private only	Go to Qu 5
- NHS & Private	Go to Qu 3
**Qu3. For NHS practices in what setting is your unit?**	
- Acute<br>- Community	
**Qu4. Please indicate the size of your unit – in terms of staffing?**	
- Consultants<br>- (Number)<br>- Podiatric Surgeons<br>- (Numbers)<br>- Registrars<br>- (Number)<br>- Trainees<br>- (Number)<br>- Staff Podiatrists<br>- (Number)<br>- MSK – Podiatrist<br>- (Number)<br>- Other<br>- (Number)<br>- (Free text – please state job role)	
**Qu5. If you are only work in private practice, would you still be willing to be involved in research?**	
- Yes<br>- No	
**Qu6. Do you have any specific time in your job plan/timetable for research and audit?**	
- Yes<br>- (number)<br>- No	

Qu7. Are you currently involved in any research / audit?
- Yes Go to Qu 8<br>- No Go to Qu 10
Qu8. If yes, please give details
- Type audit, case study, service evaluation, research<br>- Free Text<br><br>- Area / subject<br>- Free text<br><br>- Sample size<br><br>- Free text<br><br>- Staff involvement<br><br>- Free Text
Qu9. Would you be interested in collaborating your research with other centres for a larger multi centre study?
- Yes Go to Qu 11<br>- No Go to Qu 11
Qu10. If no – are there any specific obstacles preventing you from undertaking research
- Time<br>- Lack of staff<br>- Lack of resources - funding<br>- Size of caseload – too small<br>- Inexperience with research / audit<br>- Lack of research support within Trust<br>- Other – free text
Qu11. Is your Trust affiliated to an academic institution that could assist you with research?
- Yes<br>- (free text – please state where)<br>- No
Qu 12. If you were to participate in research how would you like to be involved – please tick all that apply?
- All areas<br>- Literature review<br>- Research planning & design<br>- Data Collection<br>- Statistical analysis<br>- Other – please specify free text
Qu12. Which of the following areas of research interest you? Please tick all that apply
- Improve Profile of profession<br>- Improve GIRFT<br>- Improve health economics<br>- All<br>- Other

- (Free Text – please state what)
Qu13. Are there any other areas of research that you would like to see as part of the research strategy?
- Free Text
Qu14. What would be your top 3 research areas/questions which you think podiatric surgery should try to answer?
- Free text
Qu15. Any other comments
- Free text
